# Priority setting towards achieving under-five mortality target in Africa in context of sustainable development goals: an ordinary least squares (OLS) analysis

**DOI:** 10.1186/s41256-019-0108-0

**Published:** 2019-06-26

**Authors:** Michael Acheampong, Chukwudi Ejiofor, Abraham Salinas-Miranda, Bryana Wall, Qiuyan Yu

**Affiliations:** 1 Center for Urban Ecology and Sustainability, Suffolk University, 8 Ashburton Pl, Boston, MA 02108 USA; 2 College of Public Health, University of South Florida, 13201 Bruce B. Downs Blvd. MDC 56, Tampa, FL 33612 USA; 3 College of Communications and Journalism, University of Florida, Gainesville, FL 32611 USA; 4 School of Geosciences, University of South Florida, 4202 E. Fowler Ave, Tampa, FL 33620 USA

**Keywords:** Under-five mortality, Sustainable development goals, Africa, Social determinants of health, Health literacy

## Abstract

**Background:**

Africa reduced its under-5 mortality rate (U5MR) by more than 50% during the MDGs era. However, it still has by far the highest average U5MR in the world – 81 deaths compared to a global average of 43 deaths per 1000 births, with eight of the ten countries in the world with the highest child mortality rates. The primary objective of our study was to examine the socioeconomic, healthcare, and environmental determinants that most account for U5MR disparities between African countries.

**Methods:**

We used a series of ordinary least squares (OLS) regression models to assess the effects of 14 distinct socioeconomic, environmental and healthcare variables that account for the high U5MR differentials that persist between African countries. We conducted our analysis on 43 countries for which data were available. Using a dummy variable, we also emphasized factors that may be accounting for the disparity between the eight worst-performing countries and the remainder of the continent.

**Results:**

Among all the determinants analyzed in our study, the results reveal that the factors that most account for the inequities observed are, in order, expenditure on healthcare (*p* < 0.01), total fertility rate (*p* < 0.01), income per capita (*p* < 0.05), and access to clean water (*p* < 0.1).

**Conclusions:**

Our results show that the gap between the best and worst performing countries in Africa can be significantly narrowed if government and donor interventions will target downstream factors such as improving education for mothers and sensitising them about birth control since fertility rate differences play a critical role. Improving accessibility to clean water sources to reduce outbreaks of diarrhea diseases is also observed as a critical factor.

## Introduction

Reducing child mortality is a critical objective in the Sustainable Development Goals (SDGs). The SDG target for child mortality aims to reduce under-5 mortality (U5MR) to at least as low as 25 deaths per 1000 live births [37]. While this ambitious SDG is commendable, there are concerns that without adequate prioritization of resources for the provision of maternal healthcare services, educational programs for mothers, and improving access to safe drinking water and sanitation, the new U5MR target under the SDGs may not be achieved [1, 2]. This is because previous studies [3, 7, 9, 11, 26, 36] have long established that the aforementioned factors among other socioeconomic issues are key determinants that interact to determine U5MR differences within and between countries. For the preceding Millennium Development Goals (MDGs), the under-5 mortality target was not met (MDG 4A). The UN had set a goal to cut under-five mortality rate (U5MR) down by two-thirds between 1990 and 2015, but was only able to reduce it by 53% from 91 to 43 deaths per 1000 [34] due to persistent disparities across regions and countries [4, 22, 23].

At the end of the MDGs in 2015, the United Nations Children’s Fund estimated that 5.9 million children under the age of five died globally [34]. A disproportionate number of these deaths occurred in Africa, even though like other world regions, it was able to reduce its U5MR by over 50%. In sub-Saharan Africa, approximately 1 child in 13 dies before his or her fifth birthday compared to only 1 in 189 in high-income countries **(**United Nations Inter-agency Group for Child Mortality Estimation [[32, 39]**)**. However, such regional estimates mask important disparities that exist between countries within the continent. For instance, in sub-Saharan Africa, there are huge disparities that prevail between countries [34, 38, 39]. Countries such as Liberia, Rwanda, Malawi, and Madagascar all achieved a reduction of more than 60% compared to the 1990 baseline [12, 34]. Meanwhile, according to UNICEF report, eight of the ten countries around the world where a new born is most likely to die are located in the sub-Saharan region, namely: Central African Republic (CAR), Somalia, Lesotho, Guinea-Bissau, South Sudan, Ivory Coast, Mali, and Chad [18, 33].

It is necessary to determine contextual differences between these eight worst performing countries and the rest of the continent. Acute illnesses such as malaria, diarrhea, and pneumonia among others still contribute to an inordinate amount of child deaths in sub-Saharan Africa, which can be tremendously reduced with improved antenatal and postnatal care. Although adequate investment into healthcare provision and services is still needed, action on the social determinants of health is also greatly needed to curtail child mortality in Africa, particularly the low level of education for many mothers [12, 27, 39].

Previous studies [1, 2] have examined the factors that most account for global U5MR disparities, in order to tailor intervention measures effectively to areas that need to be addressed. These studies have further illuminated that there are complexities and nuances that need deeper exploration to understand the most effectual areas for intervention to curb childhood mortality. In this regard, we argue that while understanding global disparities is important, it is critical to recognise that African countries have marked peculiarities that distinguish them from other world regions. This study, therefore, builds on them by examining and understanding key determinants – healthcare accessibility, social, economic, and environmental factors – that underlie the disparities between African countries. Finally, we sought to identify factors that most explain the gap in U5MR between the eight aforementioned UNICEF-identified worst performing countries for childhood survival and the rest of the continent. The results in the study will help interventions of policy makers and program planners to appropriately target critical and most effectual areas in Africa, as well as help narrow the gap between the worst performing countries and the rest of the countries in Africa.

## Methods

### Data and sources

**Fig. 1:**
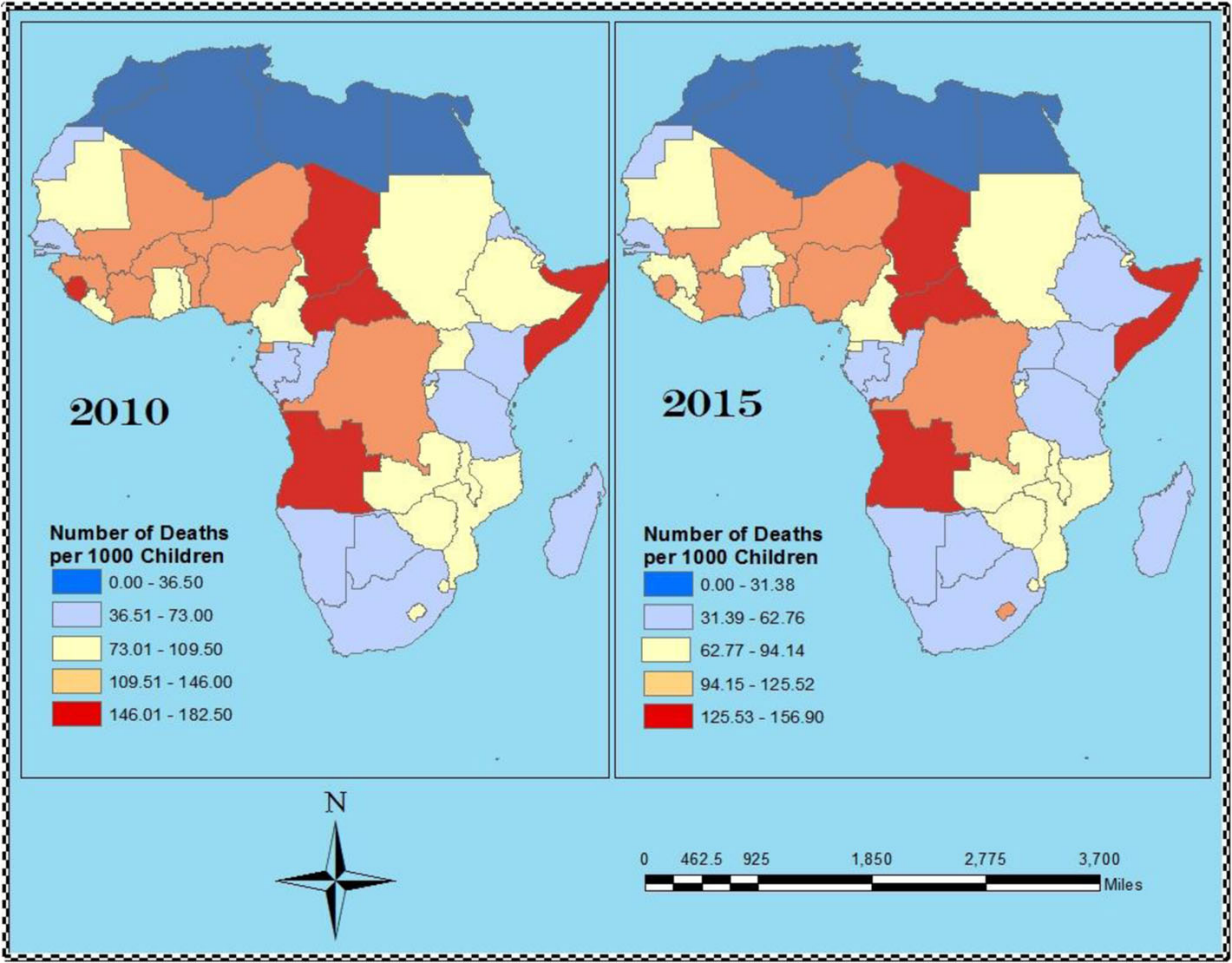
Equal Interval Distribution of Under-five Mortality Rate in Africa for 2010 (left) and 2015 (right) (data obtained from UN IGME, 2016)

As previously noted, this paper is among a series of studies carried out to identify important focal areas of intervention to reduce U5MR and builds on Acheampong et al. [1, 2] by using the similar variables but limiting the scope to intra-Africa country differentials. We utilised data from the year 2010, which was the closest year with the most comprehensive data record for all variables of interest [2]. To demonstrate that data from 2010 can provide useful insight into what prevails currently, we presented Fig. 1 (based on data obtained from UN IGME) – the equal interval distributions of U5MRs within Africa in 2010 and 2015 – which shows that the U5MR distribution in Africa has been relatively constant over the years.

All data used in this study were secondary data obtained from globally prominent databases (see Appendix A) such as the World Health Organization (WHO) Global Health Expenditure Database [37–39], World Bank’s World Development Indicators (WDI) database [31], United Nations Educational, Scientific and Cultural Organization ([35], the Central Intelligence Agency (CIA) World Factbook [8], and UN IGME [14]. While it would be ideal to analyze data for all 54 African countries, the dearth of data meant that we could carry out the analysis on 43 of the countries for which data were obtained. All eight countries identified as worst performing countries in Africa with the exception of Somalia, for which data was unavailable, were included in the study. In addition, since the data precedes South Sudan’s independence, Sudan was used as a proxy. The total list of countries considered in the study are presented in Table 1 below.

**Table 1:** 2010 under-five mortality rates (per 1000 live births) in African countries considered in this study (UN IGME, 2016)

Country	U5MR	Country	U5MR	Country	U5MR
Algeria	27.4	Gabon	63	Mozambique	103.8
Angola	182.5	Gambia	81.4	Namibia	55.4
Benin	111.6	Ghana	74.7	Niger	123.6
Botswana	60.3	Guinea	111.9	Nigeria	130.3
Burkina Faso	113.5	Guinea-Bissau	115.9	Rwanda	64.2
Burundi	98.8	Kenya	63.6	Senegal	64.8
Cameroon	106	Lesotho	101.5	Sierra Leone	160.2
Cape Verde	27.9	Liberia	89.3	South Africa	54.4
Central African Republic	150.9	Madagascar	60.3	Sudan	80.2
Chad	160.1	Malawi	92	Tanzania	62.3
Comoros	86	Mali	136.6	Togo	90.9
Congo, Dem. Rep.	116.1	Mauritania	97.8	Tunisia	17.4
Cote d’Ivoire	110.1	Mauritius	15.2	Uganda	79.5
Egypt	29	Morocco	33.1	Zambia	84.8
Equatorial Guinea	110.9				
Ethiopia	75.7				

In total, 14 variables were used as independent variables (IV) in this study, while U5MR was the dependent variable. As shown in Table 2, thirteen of the 14 variables were categorised into 4 distinct classes: healthcare accessibility, social, economic, and environmental. The fourteenth variable was a dummy variable for the seven among the eight countries in Africa currently characterised by Howard [18] to be the most dangerous countries to be born in (henceforth referred to as the UNICEF-8). The value of 1 was assigned to those countries, while 0 was assigned to the remaining 36 countries. Analysis with dummy variables served to reveal if priority factors identified as accounting for the general differences in U5MR between African countries differed when the rest of the countries compared with the UNICEF-8.

**Table 2:** Independent Variables and their Respective Classes

Variables	
Total Fertility Rate^a^	Percent Population Living under National Poverty Line^b^
Adolescent Fertility Rate^a^	Per Capita Total Expenditure on Health^c^
Total Adult Literacy Rate^a^	Out-of-pocket Expenditure as a Percent of Total Health Expenditure^c^
Female Adult Literacy Rate^a^	Government Expenditure on Health as a Percent of Total Health Expenditure^c^
Rural Population^a^	Percent Population with Access to Improved Sanitation^d^
GNI per Capita^b^	Percent Population with Access to Improved Drinking Water Source^d^
Total Female Employment to Population Ratio^b^	

Notes: ^a^ = Social; ^b^ = Economic; ^c^ = Healthcare Accessibility; ^d^ = Environmental

### Construction of models and various specifications

Ordinary least squares (OLS) regression models were used to ascertain the factors that account for inter-country U5MR differentials within Africa, as well as determine the factors that account for the differences between the UNICEF-8 and the rest of the countries on the continent. Assumptions of OLS were met by transforming data, in cases where needed, to improve linearity, normality, homogeneity of variances, and homoscedasticity [16, 24].

### Bivariate regression analysis

Before carrying out analysis based on all the variables, we conducted a simple bivariate regression analysis between U5MR and each of the 13 independent variables to examine their relationships. For each model, a second was constructed with a dummy variable for the UNICEF-8 to examine if the strength of association remained constant or changed when the UNICEF-8 are compared with the rest of the continent. The following equations were used in the analyses:

*MR* = *α* + *βnXn* + *ε*… (1)

*MR* = *α* + *βnXn* + *βU*8 + *ε…* (2)

Where:

Equation 1 = Simple bivariate analysis between each independent variable and U5MR.

Equation 2 = Simple bivariate analysis between each independent variable and U5MR, together with UNICEF-8 dummy.

*MR*= the U5MR of any given country;

*α*= the Y-intercept.

*U*8= UNICEF-8 dummy;

*β*= slope of the UNICEF-8 dummy;

*βn*= the slope associated with the predictor variable under consideration;

*Xn*= any of the 13 independent variables studied.

*ε*= the error term.

### Multivariate regression analysis

Several multivariate models were constructed to examine the factors that most account for the U5MR differentials between African countries. In order to identify which factors carried the greatest explanatory power, we constructed four models for each of the classes of variables as identified in Table 1, as well as a full model that combined all the variables. As in the case of the bivariate analyses, each multivariate regression model was constructed with a second that contained the dummy variable for UNICEF-8. The purpose of this was to determine if factors that accounted for the differences between all countries within the continent together as obtained from the first model were different from the factors that account for the differences between the UNICEF-8 countries on one hand and the remaining African countries on another. Subsequently, we dealt with issues pertaining to multicollinearity (high correlations between variables) to remove superfluous variables and aid in the ranking of the most important factors to consider. Below are the two formulas of full models with and without the UNICEF-8 dummy variable. Every other model is a subset that belongs under the full models:

*MR* = *α* + *βn*1*Xn*1 + *βn*2*Xn*2 + *βn*3*Xn*3 +  …  + *Bn*13*Xn*13 + *ε*… (1)

*MR* = *α* + *βn*1*Xn*1 + *βn*2*Xn*2 + *βn*3*Xn*3 +  …  + *Bn*13*Xn*13 + *βU*8 + *ε*… (2)

Where:

Equation 1 = Multivariate regression analysis between independent variables and U5MR.

Equation 2 = Multivariate regression analysis between independent variables and U5MR, together with UNICEF-8 dummy.

*MR*= the U5MR of any given country;

*α*= the Y-intercept;

*U*8 = UNICEF-8 dummy;

*β*= slope of the UNICEF-8 dummy;

*βn*1 … *βn*13= the slope associated with each of the 13 predictor variables studied;

*Xn*1= Total Fertility Rate.

*Xn*2= Adolescent Fertility Rate.

*Xn*3= Total Adult Literacy Rate.

*Xn*4= Female Adult Literacy Rate.

*Xn*5= Rural Population.

*Xn*6= Gross National Income per Capita.

*Xn*7= Total Female Employment to Population Ratio.

*Xn*8= Percent Population Living under National Poverty Line.

*Xn*9= Per Capita Total Expenditure on Health.

*Xn*10= Out-of-pocket Expenditure as a Percent of Total Health Expenditure.

*Xn*11= Government Expenditure on Health as a Percent of Total Health Expenditure.

*Xn*12= Percent Population with Access to Improved Sanitation.

*Xn*13= Percent Population with Access to Improved Drinking Water Source.

*ε*= the error term.

## Results

### Summary statistics of independent variables

In Table 3, we present the summary of all independent variables utilised in this study. As shown in the table, variation in total expenditure on health among African countries is the highest among all the variables considered (CV = 1.58), followed by income per capita (CV = 1.31), while access to water exhibited the least variation (CV = 0.23).

**Table 3:** Descriptive statistics for independent variables in the study

Variable^a^	Minimum	Maximum	Mean	Standard Deviation	Coefficient of Variation (Mean/SD)
Total Fertility	1.52	7.58	4.87	1.27	0.26
Adolescent Fertility	10.73	210.37	105.12	42.81	0.41
Adult Literacy	25.31	94.23	62.41	19.14	0.31
Female Literacy	12.19	92.18	54.83	22.88	0.42
Rural Percent	14.30	89.36	61.98	15.89	0.26
Income per Capita	560.00	26,790.00	4042.33	5284.64	1.31
Female Employment	11.80	86.40	57.14	18.31	0.32
Poverty Level	8.00	76.80	46.21	15.90	0.34
Gov. Expenditure	1.84	20.08	10.14	3.94	0.39
Total Expenditure	11.90	896.19	122.25	192.95	1.58
Personal Expenditure	7.45	88.15	39.03	21.07	0.54
Sanitation	9.50	92.70	34.12	21.48	0.63
Water	44.00	99.00	69.14	15.89	0.23

Note: All variables have 43 observation, i.e. total number of countries for which data was obtained

^a^Variable names are abbreviated all through the paper and provided below:

Total Fertility = Total Fertility Rate; Adolescent Fertility = Adolescent Fertility Rate; Adult Literacy = Total Adult Literacy Rate; Female Literacy = Female Adult Literacy Rate; Rural Percent = Percent Rural Population; Income per Capita = Gross National Income per Capita; Female Employment = Total Female Employment to Population Ratio; Poverty Level = Percent Population Living under National Poverty Level; Government Expenditure = Percent Government Expenditure on Health Per Capita; Total Expenditure = Total Expenditure on Health; Personal Expenditure = Percent Out-of-Pocket Expenditure; Sanitation = Percent Population with Access to Improved Sanitation Facilities; Water = Percent Population with Access to Improved Drinking Water

### Bivariate results

The results of simple regression analysis between each of the IVs and the DV are presented in Table 4 below. Unlike in Acheampong et al. [2], not all IVs demonstrated a strong association with U5MR differentials among countries in Africa. For instance, percent rural population showed no relationship with U5MR both in the general disparity analysis, and comparison between the UNICEF-8 and the rest of the continent. Likewise, the total female employment to population ratio showed no relationship with U5MR in the general analysis. However, it gained a slight increase in statistical significance when the UNICEF-8 dummy variable was included (*p* < 0.1).

**Table 4:** Results for bivariate relationships between each IV and the DV

Dependent Variable = U5MR.													
Ind. Variable													
Total Fertility	19.06*** 18.21***												
	(3.29) *(3.02)*												
Adolescent Fertility		0.57*** 0.53***											
		(0.10) *(0.09)*											
Female Literacy			−0.83*** -0.76***										
			(0.21) *(0.20)*										
Adult Literacy				−0.97*** -0.87***									
				(0.25) *(0.24)*									
Rural Percent					0.54 *0.49*								
					(0.34) *(0.32)*								
Income per Capita						−13.64* -12.10*							
						(5.33) *(5.07)*							
Female Employment							0.35 *0.49* ***.***						
							(0.30) *(0.28)*						
Poverty Level								1.03** 0.91**					
								(0.32) *(0.31)*					
Gov. Expenditure									−3.41* -2.68*				
									(1.33) *(1.32)*				
Total Expenditure										−77.64**.** *-74.34* ***.***			
										(40.82) *(38.16)*			
Personal Expenditure											3.96* *2.85* ***.***		
											(1.50) *(1.59)*		
Water												−1.09** -1.02**	
												(0.31) *(0.29)*	
Sanitation													−23.40** -19.23*
													(8.26) *(8.18)*
BP	30.69**	24.11*	29.20*	28.43*	35.13*	32.51*	40.44**	29.77*	28.99*	35.59*	*25.92* ***.***	32.61*	28.69*
	*(10.52)*	*(10.80)*	*(12.25)*	*(12.46)*	*(13.73)*	*(13.29)*	*(13.79)*	*(12.97)*	*(13.92)*	*(13.48)*	*(14.77)*	*(12.37)*	*(13.62)*
*R* ^*2*^	**0.45** ***0.52***	**0.46** ***0.52***	**0.28** ***0.37***	**0.27** ***0.35***	**0.06** ***0.19***	**0.14** ***0.25***	**0.03** ***0.20***	**0.20 ** ***0.30***	**0.14** ***0.22***	**0.08** ***0.22***	**0.15** ***0.21***	**0 .23 ** ***0.36***	**0.16** ***0.25***

Notes: Standard errors are reported in parenthesis

Entries in the table are standardised regression coefficients

Results with regression with dummy variables are presented in *italics*

Number of observations = 43

**.** *p* < 0.1

*p < 0.05

**p < 0.01

***p < 0.001

R-square values are set in boldface

### Multivariate results

In Table 5, we present the results for eight different multivariate regression models (1–8) that examines the relationship between a combination of the different IVs and U5MR. In columns 1–4, we examined the effect of the four classes of IVs as described in Table 1. Model for social variables is captured in column 1, while that of economic variables is captured in column 2. Variables of accessibility to healthcare and environmental variables are captured by columns 3 and 4, respectively. As in the bivariate analysis, each model was ran twice with and without the UNICEF-8 dummy variable, in order to identify factors that generally explain U5MR disparities between African countries, and those that are prominent in explaining the difference that exist between the UNICEF-8 countries and the rest of Africa. In column 5, results for the fully specified model is presented, while column 6 presents results for the most parsimonious subset of the models for both full models with and without the dummy variable. In columns 7 and 8, results are presented after addressing issues of multicollinearity in the most parsimonious models in column 6, respectively for that without and with the UNICEF-8 dummy variable.

**Table 5:** Results for multivariate regression models with U5MR in 2010 as DV

Dependent Variable = U5MR								
Ind. Variable	Model 1	Model 2	Model 3	Model 4	Model 5	Model 6	Model 7	Model 8
Total Fertility	11.40				9.87	10.55 *	13.57 **	
	12.35*				*10.51* ***.***	12.54 *		16.10***
	(5.71)				(5.72)	(4.95)	(4.33)	
	*(5.38)*				*(5.66)*	*(4.73)*		*(3.94)*
Adolescent Fertility	0.31*				0.19	0.19		
	*0.26* ***.***				*0.16*	*0.17*		
	(0.14)				(0.15)	(0.12)		
	*(0.13)*				*(0.16)*	*(0.12)*		
Female Adult Literacy	0.66 *0*				1.38	1.35		
	*33*				*1.00*			
	(1.18)				(1.30) *(1.31)*	(1.02)		
	*(1.11)*							
Total Adult Literacy	−0.93				−1.86	−1.73	−0.36	
	*-0.48*				*-1.46*	*-0.39*		*-0.50***.**
	(1.36)				(1.42)	(1.16)	(0.26)	
	*(1.29)*				*(1.43)*	*(0.26)*		*(0.25)*
Rural Percent	−0.11				−0.07			
	*-0.12*				*-0.08*			
	(0.30)				(0.34) *(0.34)*			
	*(0.29)*							
Income per Capita		−7.80			−20.60**.** *-17.54*	−21.73 *	−20.39*	
		*-5.55*						
		(6.47)			(11.75)	(8.78)	(8.91)	
		*(6.24)*			*(11.79)*			
Female Employment		−0.09			0.13			
		*0.11*			*0.19*			
		(0.32)			(0.26)			
		*(0.32)*			*(0.26)*			
Poverty Level		0.83 *			0.22	*0.47* ***.***		
		*0.70* ***.***			*0.17*			*0.50***.**
		(0.37)			(0.33)	*(0.26)*		
		*(0.35)*			*(0.33)*			*(0.26)*
Gov. Expenditure			−2.36		−3.46 *		−3.13**	
			*-2.36*		-3.32 *	*1.62* ***.***		*-1.79***.**
			(1.70)		(1.56) *(1.54)*	(1.02) *(0.92)*	(0.99)	
			*(1.64)*					*(0.92)*
Total Expenditure			−59.37		189.54 *	189.68 **	207.04**	
			*-67.00*		169.89 *	79.66 *		94.08*
			(41.05)		(71.08)	(62.75)	(62.98)	
			*(39.86)*		*(71.45)*	*(38.59)*		*(37.45)*
Personal Expenditure			1.57		−0.28			
			*0.29*		*-0.89*			
			(2.02)		(1.73)			
			*(2.06)*		*(1.76)*			
Water				−0.85 *	− 0.47	− 0.53**.**	−0.54**.**	
				-0.87 *	*-0.43*			
				(0.35)	(0.38)	(0.29)	(0.30)	
				*(0.33)*	*(0.38)*			
Sanitation				−13.18	2.32			
				*-8.56*	*3.33*			
				(8.85)	(10.10)			
				*(8.62)*	*(9.98)*			
BP	25.57*	30.45*	*28.04* ***.***	29.73 *	*16.15*	*17.98* ***.***		*19.21* ***.***
	*(10.46)*	*(13.57)*	*(14.44)*	*(12.70)*	*(11.74)*	(*9.84)*		*(9.90)*
Constant	31.63	118.85	201.00 *	104.28 ***	−49.24	−30.12 *-*	−76.77	
	*20.17*	*90.99*	*222.17* *	*175.67* ***	*-48.81*	*106.04*		*-123.98* **.**
	(36.70)	(67.28)	(85.78)	(28.33)	(114.36)	(75.00)	72.14	
	*(34.77)*	*(65.24)*	*(83.61)*	*(28.01)*	*(113.64)*	*(73.07)*		*(72.61)*
F-statistic	8.78 ***	4.06 *	3.58 *	7.49 **	5.21 ***	9.44***	11.32***	
	9.30 ***	*4.61* **	*3.82* *	*7.37* ***	*5.12* ***	10.87***		12.12***
*Adj. R* ^*2*^	**0.48** ***0.54***	**0.24** ***0.26***	**0.16** ***0.21***	**0.24** ***0.31***	**0.57** ***0.58***	**0.62** ***0.62***	**0.60**	***0.61***

Notes: Standard errors are reported in parenthesis

Entries in the table are standardised regression coefficients

Results with regression with dummy variables are presented in *italics*

Number of observations = 43

**.** p < 0.1

*p < 0.05

**p < 0.01

*** *p* < 0.001.

R-square values are set in boldface

From the results in columns 1–4 in Table 5, it is seen that among the 43 countries studied in Africa, all classes of independent variables are associated with the variability in U5MR across countries in the sub-region. This shows that there is a combination of factors from different classes that account for the variability. From the table, adjusted R^2^ for first model (without dummy variable) in column 1 indicated that 48% of the differences in U5MR among African countries can be attributed to social factors. Within the model, only Total Fertility Rate (*p* < 0.1) and Female Adult Fertility Rate (0 < 0.05) demonstrated significant positive associations with U5MR. All other variables within this class were not significant. In the second model in the column (with dummy variable), the explanatory power of social factors for U5MR differentials increased to 54%, with a significant positive dummy variable (*p* < 0.05) that demonstrates that social factors greatly account for the U5MR differentials between the UNICEF-8 and the rest of the continent. It is also worth noting that Total Fertility Rate (*p* < 0.05) increased in significance, while Female Adult Fertility Rate (0 < 0.1) decreased in significance when the dummy variable was considered. In the column 2, adjusted R^2^ for first model was 0.24. Within the model, only Percent Population Living under National Poverty Line showed a strong positive relationship with the DV (*p* < 0.05). Both GNI per capita and Total Female Employment to Population Ratio were not statistically significant. In the second model in the column (with dummy variable), the explanatory power of economic factors for U5MR differentials remained relatively constant, with a significant positive dummy variable (p < 0.05). This demonstrates that economic factors greatly account for the U5MR differentials between the UNICEF-8 and the rest of the continent, even though Percent Population Living under National Poverty Line decreased in significance (*p* < 0.1).

Adjusted R^2^ for first model in column 3 showed that only 16% of the differences in U5MR among African countries can be attributed to healthcare accessibility factors. In this model, none of the variables was significant. In the second model in the column (with dummy variable), the explanatory power of healthcare accessibility factors for U5MR differentials increased to 21%, with a significant positive dummy variable (p < 0.1). However, similar to the first model, none of the variables showed statistical significance. The R^2^ in column 4, showed that environmental variables as classified in Table 1 can explain 24% of the differences in U5MR among African countries. The model showed that only Percent Population with Access to Improved Drinking Water Source showed a strong negative relationship with U5MR (*p* < 0.05). Percent Population with Access to Improved Sanitation was not statistically significant. In the second model in the column (with dummy variable), the explanatory power of environmental factors for U5MR differentials increased to 21%, with a significant positive dummy variable (p < 0.05). This demonstrates that environmental factors greatly account for the U5MR differentials between the UNICEF-8 and the rest of the continent, with Percent Population with Access to Improved Drinking Water Source remaining constant.

In Table 6 below, we rank order the t-statistic of the UNICEF-8 dummy variable as obtained in columns 1–4 to demonstrate which of the various classes has the highest explanatory power for the U5MR gap between the UNICEF-8 and the rest of the continent. The table shows that that the class of social factors (2.44) has the strongest effect on difference in U5MR between the UNICEF-8 and the rest of the continent. Environmental factors (2.34) was second on the list. Ranking third was the class of economic factors (2.24), while healthcare access (1.94) ranked fourth.

**Table 6:** Rank Ordering Classes of Variables

Ind. Variable	*β*	SE	*t*-statistic
UNICEF-8*Social	25.57	10.46	2.44 *
UNICEF-8*Environmental	29.73	12.70	2.34 *
UNICEF-8*Economic	30.45	13,57	2.24 *
UNICEF-8*Healthcare Access	28.04	14.44	1.94**.**

Number of observations = 43

**.** *p* < 0.1

*p < 0.05

Column 5 contains the two full models, which yielded similar results albeit weakly as seen from the F-statistics of 5.21 and 5.12 (*p* < 0.001). Together, all the variables account for nearly 60% of the variability in U5MR across countries in Africa, with adjusted R^2^ values of 0.57 and 0.58 for the first and second models, respectively. In the first model, Total Fertility Rate (*p* < 0.1) and Per Capita Total Expenditure on Health (*p* < 0.05) were the only variables that exhibited positive associations, while Gross National Income per Capita (p < 0.1) and Government Expenditure on Health as a Percent of Total Health Expenditure (p < 0.05) were the only variables with a negative association with U5MR. In the second model, all the variables maintained their significance except for Gross National Income per Capita that became statistically insignificant. It is important to observe that the UNICEF-8 dummy variable was not statistically significant in the column, meaning that when considered together, the variables do not explain the difference in U5MR between the UNICEF-8 countries and the rest.

In column 6, the results of the most parsimonious models are presented. The two models (with and without the dummy variable) were stronger than the full model and yielded similar results, both with the ability to explain about 62% of U5MR differentials. There was, however, differences found in the variables of importance in the two models. In the first model, Total Fertility Rate (*p* < 0.05), and Per Capita Total Expenditure on Health (*p* < 0.01) were the only variables that exhibited positive associations, while Government Expenditure on Health as a Percent of Total Health Expenditure (*p* < 0.05), Gross National Income per Capita (p < 0.01) and Percent Population with Access to Improved Drinking Water Source (*p* < 0.1) were the only variables with a negative association with U5MR. In the second model, Total Fertility Rate (p < 0.05), Percent Population Living under National Poverty Line (*p* < 0.1) and Per Capita Total Expenditure on Health (p < 0.05) were the only variables that exhibited positive associations, while Government Expenditure on Health as a Percent of Total Health Expenditure (p < 0.1) was the only variable with a negative association with U5MR. It is important to observe that the UNICEF-8 dummy variable was statistically significant (p < 0.1) in the column, meaning that the variables have a significant explanatory power for the U5MR differentials between the UNICEF-8 countries and the rest. One of the most important observations in the full and parsimonious models is that the relationship between Per Capita Total Expenditure on Health and U5MR changed from negative in the bivariate analysis to a statistically significant positive relationship, when interacting with all other variables. This demonstrates the complexity and nuances that can exist in understanding factors that contribute to U5MR differences between countries.

Columns 7 and 8 contain the results of single models for the most parsimonious models without and with the dummy variables, respectively, after addressing issues of multicollinearity. After the variance inflation factor (VIF) analysis, we dropped variables of lower strength that decreased the significance of other variables in the models because they have a high correlation. In the column 7 model, we dropped Adolescent Fertility Rate and Female Adult Literacy. The explanatory power of the model remained relatively constant, accounting for about 60% of the U5MR differentials between African countries. However, as it can be noticed, Total Adult Fertility increased in its significance (from *p* < 0.05 to *p* < 0.01), while other variables maintained their significance from the most parsimonious model. In the column 7 model, we also dropped Adolescent Fertility Rate. In this case, as in the previous case, the explanatory power of the model remained relatively constant, with an adjusted R^2^ of 0.61. The significant positive dummy variable indicates that there is an increasing level of U5MR among the UNICEF-8 countries, compared with the rest of the countries that can be explained by differences that exist in variables such as Total Fertility Rate, Total Adult Literacy, Percent Population Living under National Poverty Line, Government Expenditure on Health as a Percent of Total Health Expenditure, and Per Capita Total Expenditure on Health. It can also be noticed that while all variables maintained their level of significance Total Adult Fertility increased in its significance (from *p* < 0.05 to *p* < 0.001) and Total Adult Literacy gained significance (*p* < 0.1).

In Table 7 above, we presented a ranking of the significant variables in models from columns 7 and 8 as first and second model, respectively. This ranking is based on the absolute values of associated *t*-statistics, and represents their order of importance on U5MR. In the first model (without dummy variable), it shows that Per Capita Total Expenditure on Health on the U5MR differentials among African countries, followed by Government Expenditure on Health as a Percent of Total Health Expenditure. Total Fertility Rate, Gross National Income per Capita, and Percent Population with Access to Improved Drinking Water Source followed in that order. In second model (with dummy variable), it ranks the significant variables that explain the U5MR gap between the UNICEF-8 and the rest of the African countries in the following order of importance: Total Fertility Rate, Per Capita Total Expenditure on Health, Total Adult Literacy, Government Expenditure on Health as a Percent of Total Health Expenditure, and Percent Population Living under National Poverty Line.

**Table 7:** Rank Ordering of Variables

First Model		Second Model	
Ind. Variable	*t*-statistic	Ind. Variable	*t*-statistic
Total Expenditure	3.29 **	Total Fertility	4.09 ***
Government Expenditure	3.16 **	Total Expenditure	2.51 *
Total Fertility	3.13 **	Total Adult Literacy	1.98 **.**
Income per Capita	2.29 *	Government Expenditure	1.94 **.**
Water	1.84 **.**	Poverty	1.90 **.**

**.** *p* < 0.1

*p < 0.05

**p < 0.01

***p < 0.001

## Discussion

### The primacy of social factors in the U5MR disparities discourse

In the analyses presented above, it is clear that understanding the U5MR disparities among African countries is nuanced and multidimensional, as there is a combination of important factors that belong to different classes, as was observed by Acheampong et al. [2]. The findings of this study, however, emphasise the argument that regional priorities may differ drastically from the global as the factors identified in this study as critical to explaining the U5MR differentials within Africa are different. From the Table 5, gap in social factors account most for U5MR disparities. This means that when considered separately, addressing issues pertaining to social factors, in theory, will be the most efficient approach to closing the gap in U5MR. In the table, it is obvious that the gap in number of child births directly relates to gap in U5MR. While this holds true for the general disparities around the continent, it is even more prominent for differences between UNICEF-8 countries and the rest of the continent. For this reason, education on birth control and/or family planning would be critical to close the mortality gap on the continent, especially if the prime focus is to elevate performance of the UNICEF-8 countries.

### Understanding the nuances in effect of health expenditure

One of the most critical findings in this study that highlights the complex nuances inherent in the dynamics of U5MR pertains to Total Health Expenditure per Capita and Government Expenditure on Health. While the two variables individually showed a negative association with U5MR across countries around the continent (Table 4), the relationship of Total Health Expenditure reversed after interacting with other factors. As a matter of fact, it shows that in Africa, high expenditure on healthcare per capita is the most important factor that explains high mortality rate of children under 5 years old in countries. While this finding can be curious, the fact that the government expenditure have the opposite effect lends a possible interpretation. This combined with the importance of access to clean water in the ranking of factors in the first model in Table 7 can provide basis for speculation.

Total expenditure on health is a broader variable that incorporates health expense from all sources – personal, government, and donor, among others. According to Table 3, this is the factor for which African countries showed the greatest difference. Yet, it has the worst impact on under-5 mortality (Table 5). Meanwhile, in government expenditure on health, there was not as much differences between African countries (Table 3) and yet showed a positive impact of under-5 mortality (Table 5). Countries with relatively better economies are most likely to accommodate most of their health expenditure on the government level [10, 21], as well as be able to provide more access to better quality drinking water due to the associated high capital cost [19]). [34]) has indicated that 90% of total diarrhea deaths in children, which can be drastically reduced with access to clean drinking water, occur in sub-Saharan Africa. This means that countries that are able to provide greater access to clean water will cut down the number of outbreaks and limit the need for external support. On the other hand, during the 2011 cholera outbreaks in West and Central Africa, there were about 2500 children lives that were claimed, most of them under the age of five [20]. Such epidemics in developing regions usually elicit international response ([5] [29]), which helps reduce the number of casualties, but not until it has drastically increased the total expenditure on health per capita. This is because countries that receive medical assistance from foreign professionals are most likely to record higher cost per head than countries that do not require such, due to disparity of salaries and similar other factors between donor and recipient countries. This finding reveals that intervention in more downstream factors such as investing in communities to improve their conditions of life may reduce burden of incurring higher costs of intervening in times of outbreaks, but with limited success [15, 28].

### The role of literacy

Another important factor that surfaces within the African continent is the role of literacy rate, as seen in the second model in Table 7. In the quest to narrow the U5MR gap between the UNICEF-8 countries and the rest of the continent, it is important to recognise that investment in education will be pertinent. This finding is not surprising as studies like those conducted by Breierova and Duflo [6] and [25]) have demonstrated that higher parental education associates negatively with child mortality. This is because the higher education achieved, the more knowledgeable parents are about pre-and-post-natal healthcare. Additionally, educated parents are less likely to be poor, as well as, less likely to have many children because studies have found that with more education, women are more likely to delay child birth and have fewer children [17, 30].

### Study’s implications for policies and interventions

The findings of this study supports the argument that it is important to under regional peculiarities when drawing global agenda and associated goals. It has revealed that priorities to address global U5MR differentials as in Acheampong et al. [2] may not necessarily be the same as those that require attention in addressing intra-regional disparities in Africa. Since most African countries share similar characteristics, addressing gap areas between them may not present as much a challenge as addressing gaps that exist between them and countries from other parts of the world with whom they share very little in common.

The study has shown that government and donor interventions will be more effectual should they be proactive target downstream factors such as improving educating mothers and sensitizing them about birth control since fertility rate differences greatly determine the difference in child mortality between countries. When interventions are knee-jerk and reactive, such as shipping medical personnel and medication from donor countries to contain outbreaks, the study indicates that they come with significantly higher cost but end up doing little to improve the situation. Another critical area that will help reduce the U5MR in Africa over the long term is improving accessibility to clean water sources, which is pertinent to reduce outbreaks of diarrhea diseases that are responsible for claiming an inordinate amount of child lives in Africa.

### Study limitations

This study has several limitations that need to be acknowledged. A number of these limitations revolve around data availability. While this study utilizes as comprehensive data as reliable, and identifying with the shortcomings of Acheampong et al. [2] as this work builds on its findings and uses similar variables and principles, it is important to reiterate the limitations. First, it is important to note that not all African countries were included in this study due to limited availability of data. Therefore, the 43 countries considered in this study are a convenient sample. However, it is the hope of the authors that 43 out of 54 countries can paint a general picture of the continent at large.

In addition, as previously noted, we made a decision to use 2010 as our reference year for this study because it is the closest year that contained most comprehensive data for most of the variables considered, as data many of the 14 variables were lacking. Even though we obtained the U5MR data for 2015, we deemed it important to use the 2010 to ensure alignment of data to understand the most important determinants of U5MR for the year 2010.

Another limitation of using 2010 data is that the UNICEF-8 countries are based on a UNICEF report from 2018 that is based on data for newborn mortality rate from 2016. This means that ranking of new born mortality rates in 2016 does not necessarily align with U5MR ranking in 2010. However imperfect the alignment, it is critical to note that many of these countries were still some of the worst performers in U5MR in 2010, which made our analysis useful. For instance, Somalia, Chad, Mali, and Central African Republic were all in the top 8, while countries such as Guinea-Bissau, Ivory Coast, and South Sudan were not far behind. This helped to understand how the importance of some determinants may shift if those countries were isolated.

As far as U5MR data is concerned, different sources provided different estimates. For this reason, we elected to use data available from the UN IGME. It is also critical to acknowledge that different sources utilize different data collection methods to generate data, which are accompanied by high levels of uncertainties. However, addressing data generation methods by the different sources for all the 14 variables considered in this research was beyond the scope of this study.

Besides the limitations related to data availability, there were other important limitations regarding variable selection and analytical decisions. For instance, it is important to acknowledge that upstream factors considered in this study may diminish the critical role of some downstream factors. However, considering that an uncountable number of factors can influence the mortality rate in children, and coupled with the fact that this is a cross-national analysis, the authors consider using such broadly defined variables very useful. This is because such broad variables can capture the essence of many downstream variables. For instance, downstream factors such as “number of births attended by skilled health personnel” and “number of hospital beds per thousand people” among many others can be viewed as important factors in understanding childhood mortality, we believe that an upstream variable such as “total health expenditure” can help condense the essence of such myriad of variables into one.

The effect of coarse variables on the outcome of the analysis is also important to mention. As Acheampong et al. [2] acknowledged, literacy rate may not have had as much significance in this study because of its broad definition. It does not account for different levels of education, as subpopulations with middle school education are typically lumped together with those with tertiary education. In reality, however, it is expected that the difference in know-how between these two groups and their ability to access and understand healthcare will be critical to the survival of their children, as many in-country studies have found [7, 9, 13]. For this reason, we expect that the availability of a disaggregated data that distinguish between people with primary, secondary and tertiary education might produce different results [13].

## Conclusion

Globally, the U5MR goal set in MDGs proved elusive even though significant progress was achieved. In Africa, the story was similar with some countries being able to meet their target. However, the continent still lags behind the rest of the globe in terms of the number of deaths recorded in children under five years old. In fact, eight of the ten countries (UNICEF-8) where it is most dangerous to be a new born are in Africa. While studies have addressed important factors that merit consideration on a global level as the world pursues the new goal in SDGs by cutting mortality down to 25 deaths per 1000 births, there is a need to acknowledge that bridging intra-continental gaps in Africa may be more realistic in the interim. This is especially because countries on the continent share many characteristics, including cultural, social, and economic. This paper has built on previous studies by understanding that generally account for intra-continental disparities between African countries and the factors that need focus to draw up the UNICEF-8 countries.

The study revealed that gap in number of child births significantly account for the gap in child deaths on the African continent. It is therefore critical to educate mothers on issues pertaining to birth control and/or family planning. This was found to be even more critical to bridge the gap between the UNICEF-8 and the rest of their counterparts on the continent. The study also provide an indication that the conventional interventions for epidemics may come with significant financial costs, while doing little to reduce the overall burden of child deaths on the continent. Rather, long-term interventions in more downstream factors such as investing in communities to improve their education and conditions of life may be more effectual.

## Data Availability

All data generated or analysed during this study are included in this published article.

## Appendix A

**Table:**

Variable	Source
U5MR	UN Inter-agency Group for Child Mortality Estimation database (https://childmortality.org/data)
Gross National Income per Capita	World Development Indicators (WDI) database (http://data.worldbank.org/indicator)
Total Fertility Rate	World Development Indicators (WDI) database (http://data.worldbank.org/indicator)
Adolescent Fertility Rate	World Development Indicators (WDI) database (http://data.worldbank.org/indicator)
Total Female Employment to Population Ratio	World Development Indicators (WDI) database (http://data.worldbank.org/indicator
Percent Rural Population	World Development Indicators (WDI) database (http://data.worldbank.org/indicator)
Percent Population with Access to Improved Sanitation Facilities	World Development Indicators (WDI) database (http://data.worldbank.org/indicator)
Percent Population with Access to Improved Drinking Water	World Development Indicators (WDI) database (http://data.worldbank.org/indicator)
Per Capita Total Expenditure on Health	WHO Global Health Expenditure Database (http://apps.who.int/nha/database/Select/Indicators/en)
Out-of-pocket Expenditure as a Percent of Total Health Expenditure	WHO Global Health Expenditure Database (http://apps.who.int/nha/database/Select/Indicators/en)
Government Expenditure on Health as a Percent of Total Health Expenditure	WHO Global Health Expenditure Database (http://apps.who.int/nha/database/Select/Indicators/en).
Percent Population Living under National Poverty Line	World Development Indicators (WDI) (http://data.worldbank.org/indicator). and the CIA world Factbook (https://www.cia.gov/library/publications/resources/the-world-factbook/)
Female Adult Literacy Rate	UNESCO (http://data.uis.unesco.org/Index.aspx?queryid=166); WDI (http://data.worldbank.org/indicator); and CIA World factbook (https://www.cia.gov/library/publications/resources/the-world-factbook/).
Total Adult Literacy Rate	UNESCO (http://data.uis.unesco.org/Index.aspx?queryid=166); WDI (http://data.worldbank.org/indicator); and CIA World factbook (https://www.cia.gov/library/publications/resources/the-world-factbook/).

**Table 8:** Dataset for the Study

	Female Literacy	Adult Literacy	Gov. Expenditure	U5MR	Total Expenditure	Personal Expenditure	Water	Total Fertility	Sanitation	Adolescent Fertility	Income per Capita	Female Employment	Rural Percent
Algeria	64.0	72.6	8.1	27.4	178.2	20.9	83.0	2.8	86.6	10.7	12570.0	11.8	32.5
Angola	58.6	70.4	7.2	182.5	123.2	17.5	51.0	6.2	46.2	180.9	5700.0	58.1	59.9
Benin	18.0	28.7	9.6	111.6	31.0	46.8	75.0	5.1	17.3	96.2	1590.0	66.4	58.1
Botswana	85.6	85.1	17.0	60.3	614.6	8.1	96.0	2.8	60.6	43.1	12300.0	56.1	43.8
Burkina Faso	21.6	28.7	13.5	113.5	39.8	36.2	79.0	5.9	17.4	121.1	1430.0	75.4	74.3
Burundi	84.5	86.9	8.1	98.8	20.7	37.9	72.0	6.3	46.9	31.6	690.0	76.8	89.4
Cameroon	64.8	71.3	8.5	106.0	61.3	66.5	77.0	5.0	44.7	121.8	2490.0	60.6	48.5
Cape Verde	80.3	84.9	10.1	27.9	154.6	24.9	88.0	2.4	64.2	79.0	5590.0	46.6	38.2
Central African Republic	44.2	56.6	8.5	150.9	18.2	61.4	67.0	4.6	20.7	101.6	870.0	67.2	61.2
Chad	25.4	35.4	3.3	160.1	30.6	72.5	51.0	6.6	11.6	164.0	1820.0	59.4	78.0
Comoros	70.6	75.5	13.1	86.0	33.2	32.8	95.0	4.9	32.7	76.5	1360.0	32.2	72.1
Congo, Dem. Rep.	46.1	61.2	9.1	116.1	15.8	35.9	45.0	6.3	26.8	126.6	560.0	64.3	60.1
Cote d'Ivoire	47.6	56.9	5.1	110.1	59.7	77.5	80.0	4.9	20.9	136.1	2570.0	50.1	49.4
Equatorial Guinea	91.1	94.2	7.0	110.9	896.2	22.2	51.0	5.1	75.8	117.7	26790.0	74.6	60.8
Eritrea	59.0	68.9	3.6	55.6	11.9	51.8	61.0	5.0	14.6	67.0	1290.0	73.6	79.4
Ethiopia	28.9	39.0	13.5	75.7	15.7	37.2	44.0	4.9	21.7	73.7	1050.0	71.6	82.7
Gabon	85.6	89.0	6.6	63.0	302.1	47.1	87.0	4.2	40.9	116.2	13950.0	40.2	14.3
Gambia	41.9	51.1	11.3	81.4	26.1	23.8	89.0	5.8	58.8	115.9	1570.0	66.9	43.7
Ghana	65.3	71.5	12.1	74.7	67.0	26.9	86.0	4.1	13.7	70.5	2970.0	64.0	49.3
Guinea	12.2	25.3	1.8	111.9	23.0	88.1	74.0	5.2	17.9	149.7	1040.0	64.5	65.1
Guinea-Bissau	42.1	55.3	4.1	115.9	46.9	66.4	64.0	5.1	18.9	108.4	1300.0	63.1	54.8
Kenya	66.9	72.2	7.3	63.6	36.8	42.7	59.0	4.6	29.2	96.6	2480.0	55.0	76.4
Lesotho	85.0	75.8	13.4	101.5	108.9	16.4	78.0	3.2	28.5	91.7	2590.0	42.7	75.2
Liberia	27.0	42.9	11.1	89.3	29.2	35.2	73.0	5.0	15.6	127.2	590.0	55.7	52.2
Madagascar	61.6	64.5	14.7	60.3	15.9	27.1	46.0	4.7	11.4	127.4	1350.0	83.1	68.1
Malawi	51.3	61.3	14.2	92.0	25.6	11.1	83.0	5.6	38.8	146.7	720.0	77.6	84.5
Mali	20.3	31.1	10.6	136.6	31.7	53.2	64.0	6.8	22.4	178.6	1440.0	44.6	64.0
Mauritania	52.0	58.6	7.3	97.8	42.7	44.3	50.0	4.8	35.7	84.4	3190.0	20.3	43.3
Mauritius	86.7	88.8	9.8	15.2	448.9	51.7	99.0	1.5	92.7	31.4	15470.0	37.8	59.4
Mozambique	36.5	50.6	12.2	103.8	21.3	13.7	47.0	5.4	18.8	161.7	870.0	78.2	69.0
Namibia	78.4	76.5	12.1	55.4	361.3	7.4	93.0	3.2	32.2	79.8	7890.0	43.1	58.4
Niger	15.1	28.7	11.1	123.6	18.3	41.3	49.0	7.6	9.5	210.4	800.0	38.0	82.4
Nigeria	41.4	51.1	4.4	130.3	62.8	59.2	58.0	6.0	30.5	119.5	4750.0	44.4	56.5
Rwanda	61.5	65.9	20.1	64.2	55.5	22.2	65.0	4.8	57.2	34.1	1290.0	86.4	76.0
Senegal	38.7	49.7	11.6	64.8	58.5	35.0	72.0	5.1	45.1	90.5	2110.0	57.1	57.8
